# ATP amplifies NADPH-dependent and -independent neutrophil extracellular trap formation

**DOI:** 10.1038/s41598-019-53058-9

**Published:** 2019-11-12

**Authors:** Aderonke Sofoluwe, Marc Bacchetta, Mehdi Badaoui, Brenda R. Kwak, Marc Chanson

**Affiliations:** 1 Department of Paediatrics, Gynaecology & Obstetrics, Geneva University Hospitals, Geneva, Switzerland; 2 Department of Cell Physiology & Metabolism, University of Geneva, Faculty of medicine, Geneva, Switzerland; 3 Department of Pathology & Immunology, University of Geneva, Faculty of medicine, Geneva, Switzerland

**Keywords:** Ion channel signalling, Neutrophils

## Abstract

Neutrophils are the first immune cells to kill invading microbes at sites of infection using a variety of processes, including the release of proteases, phagocytosis and the production of neutrophil extracellular traps (NETs). NET formation, or NETosis, is a specific and highly efficient process, which is induced by a variety of stimuli leading to expulsion of DNA, proteases and antimicrobial peptides to the extracellular space. However, uncontrolled NETosis may lead to adverse effects and exert tissue damage in pathological conditions. Here, we show that the ATP channel pannexin1 (Panx1) is functionally expressed by bone marrow-derived neutrophils (BMDNs) of wild-type (WT) mice and that ATP contributes to NETosis induced *in vitro* by the calcium ionophore A23187 or phorbol 12-myristate 13-acetate (PMA). Interestingly, neutrophils isolated from *Panx1*^−/−^ mice showed reduced and/or delayed induction of NETosis. Brilliant blue FCF dye (BB-FCF), a Panx1 channel inhibitor, decreased NETosis in wild-type neutrophils to the extent observed in *Panx1*^−/−^ neutrophils. Thus, we demonstrate that ATP and Panx1 channels contribute to NETosis and may represent a therapeutic target.

## Introduction

Neutrophils mature in the bone marrow, where they synthesise and package enzymes and antimicrobial proteins into secretory granules that are rapidly released at sites of infection and inflammation^1^. Among several antimicrobial defence mechanisms, the externalisation of decondensed nuclear chromatin was first described to capture and trap invading pathogens and facilitate their killing^2^. The formation of neutrophil extracellular trap (NET), also known as NETosis, is triggered by pathogen-associated virulence factors and pro-inflammatory stimuli^2–4^. Recent studies have provided insights into the signalling pathways and the characteristics of different forms of NETosis triggered by different stimuli. Suicidal NETosis is commonly observed in inflammatory diseases such as rheumatoid arthritis, lupus and vasculitis in which autoantibodies target released NETs^5^. Suicidal NETosis is a slow process that eventually leads to cell death through the generation of free reactive oxygen species (ROS) that activate peptidyl arginine deiminase type IV (PAD4), myeloperoxidase-dependent nuclear envelope degradation and subsequent release of nuclear and cellular contents^5,6^. NETs were originally observed in response to the protein kinase C activator phorbol 12-myristate 13-acetate (PMA), which induces NETosis through the ROS generating NADPH oxidase complex that contributes to the disruption of the extracellular membrane^6,7^. In contrast, vital NETosis is a faster and robust, NADPH-independent, process dominated by a rise in intracellular calcium concentration and often observed during infection^8,9^. A third type of very fast NETosis is characterised by the release of mitochondrial DNA through the recognition of granulocyte-macrophage colony-stimulating factor or lipopolysaccharide^10^. It is believed that end-stage of NETosis requires PAD4 citrullination of histone H3, which could play a major role in formation and/or function of NETs^11,12^. Recently, a biophysical model for NETosis was proposed whereby, independent of the stimulus, entropic swelling of the chromatin represents the main physical force leading to rupture of the nuclear envelope and the plasma cell membrane^13^. This is important because the model predicts that a point of no return is reached once NETosis is initiated.

Through multiple trigger mechanisms, excessive NETosis may exert tissue damage and enhance the severity of pathological conditions^12^. For instance, NETs are detected in multiple organs in sepsis, and may enhance inflammation and viscosity of sputum in cystic fibrosis^14,15^. NETs were shown to be present in the thrombus in deep vein thrombosis and to be targets of autoantibodies in autoimmune diseases like rheumatoid arthritis and systemic lupus erythematosus^3,5^. Thus, there is a need to identify NET inhibitors and new therapeutic targets to prevent worsening conditions in NETs-related diseases.

Accumulating evidence shows that innate immune responses are associated with extracellular nucleotides, particularly adenosine 5′-triphosphate (ATP). ATP acts as an extracellular signalling molecule that can modulate cellular processes through activation of specific cell membrane purinergic receptors. Under inflammatory conditions, ATP is present in the extracellular space due to its release from the cell cytoplasm. Among other mechanisms, pannexin-made membrane channel might be essential for inside out signalling by releasing ATP to the extracellular space^16^. Pannexin (Panx) family consists of three isoforms (Panx1, Panx2 and Panx3), Panx1 being ubiquitously expressed^17,18^. Neutrophils were found to release ATP through Panx1 channels, which in turn activates purinergic receptors in a paracrine manner^19,20^. In this study, we questioned whether ATP and Panx1 contributed to NETosis using wild-type (WT) and *Panx1*^−/−^ murine neutrophils activated with the calcium ionophore A23187 or PMA. We report here that extracellular ATP amplifies NETosis through purinergic stimulation. We also show that NETosis is reduced in *Panx1*^−/−^ neutrophils and that pharmacological inhibition of Panx1 slowed NETosis triggered by A23187 and PMA.

## Results

### NET formation induced by A23187 and PMA

**Figure 1:**
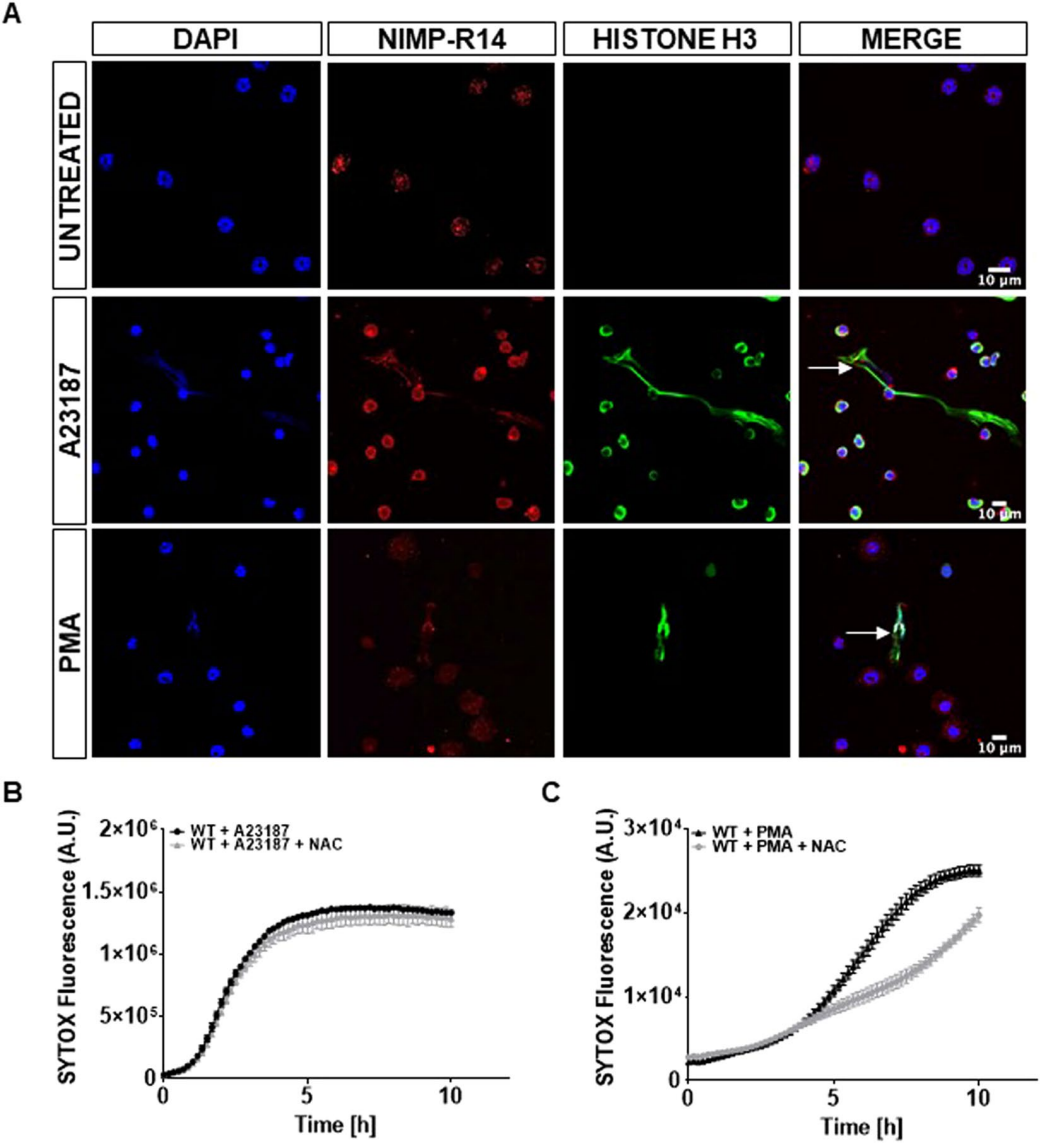
Qualitative and quantitative assessment of NETosis induced with NADPH-dependent and -independent stimuli. (**A**) Immunofluorescence staining of NETs after 2 h stimulation revealed histone H3 (histone citrullination) in green. Neutrophils were stained with the specific marker NIMP-R14 (in red) and nuclei with DAPI (blue). Untreated BMDNs (upper panels), BMDNs stimulated with 1 μM A23187 (middle panel) and with 50 nM PMA (bottom panel) are shown. White arrows indicate neutrophils with released extracellular traps. (**B**,**C**) Kinetics of SYTOX green fluorescence detection of NETosis in response to 1 μM A23187 (**B**) and 50 nM PMA (**C**), in the absence or presence of 10 mM N-acetyl-cysteine (NAC). NAC was used to inhibit reactive oxygen species.

A23187 and PMA are commonly used to trigger vital (NADPH-independent) and suicidal (NADPH-dependent) NETosis. Formation of NET was first verified by immunostaining of citrullinated histones in WT bone marrow derived neutrophils (BMDNs) exposed to A23187 or PMA for 2 h or untreated. As illustrated in Fig. 1A and Supplemental Fig. 1, histone citrullination was stronger in A23187 stimulated neutrophils when compared to PMA stimulated cells, which is consistent with the fast and robust response evoked by the calcium ionophore^21^.

NET formation can also be monitored as a function of time by using the cell impermeable SYTOX green dye, which binds to extracellular DNA. An example of NET formation by time-lapse imaging in a global population of stimulated BMDNs is shown in Video 1 (Supplemental Video 1). To provide quantitative and temporal analysis of NETosis, SYTOX green dye fluorescence over time was quantified using a plate reader. Typically, increase in SYTOX green fluorescence followed a sigmoidal curve; A23187 induced rapid and robust extracellular DNA release, reaching a plateau within 3–4 h (Fig. 1B; black curve). NET formation induced by PMA was slower and reached maximal extracellular DNA after 4–6 h (Fig. 1C; black curve). As expected, BMDN incubation with the antioxidant N-acetyl cysteine (NAC) strongly reduced the induction of NADPH-dependent NETosis while it was inefficient in modulating NADPH-independent NETosis (Fig. 1B,C; grey curves), confirming the specificity of the signalling induced in neutrophils by A23187 and PMA.

### Extracellular ATP contributes to NETosis

**Figure 2:**
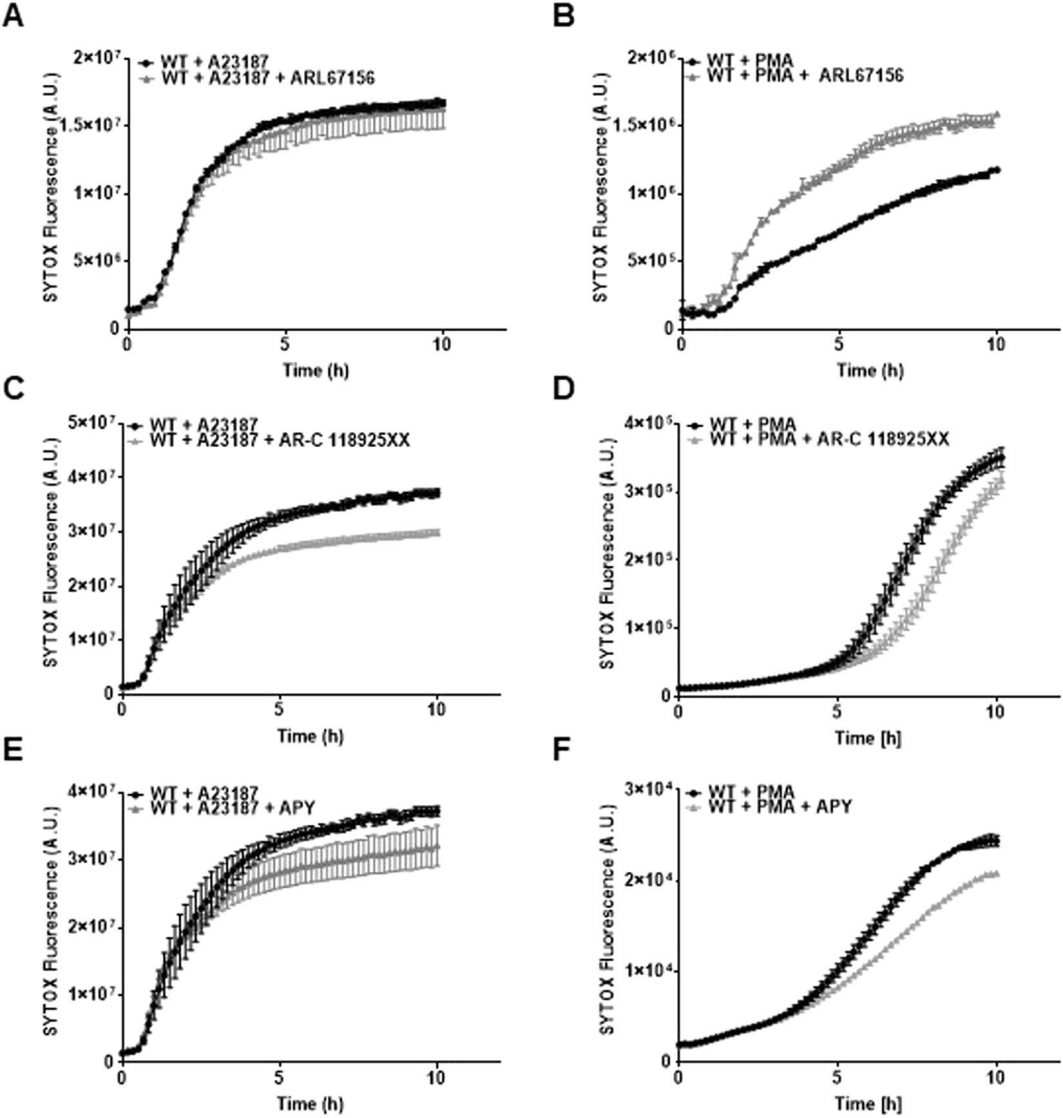
Modulation of NETosis by extracellular ATP. (**A**,**B**) Kinetics of ecto-ATPase inhibition using ARL67156 (100 μM) in A23187 and PMA stimulation respectively. (**C**,**D**) Kinetics of P2Y_2_ receptor signalling inhibition using P2Y_2_ receptor antagonist AR-C 118925XX (500 nM) in A23187 and PMA stimulation respectively. (**E**,**F**) Kinetics of ATP scavenging using 50 U/ml apyrase (APY) in A23187 and PMA stimulation respectively. Note in B that the initial phase is not shown. N = 3, n = 6.

To evaluate the contribution of ATP and purinergic signalling to NETosis, neutrophils were stimulated with A23187 or PMA in the presence of drugs that would reduce ATP degradation, such as an inhibitor of the surface ecto-enzyme CD39 (ARL67156), or drugs that would inhibit activation of purinergic receptors. As shown in Fig. 2A,B, PMA-induced, but not A23187-induced, NETosis was enhanced in the presence of ARL67156. NADPH-independent NETosis may not be enhanced by ARL67156 due to the already maximal effect caused by the calcium ionophore. In agreement, we found that a P2Y_2_ receptor antagonist (AR-C 118925XX) decreased NET formation induced by A23187 and PMA (Fig. 2C,D). These results were corroborated by treating cells with apyrase, an ATP scavenger, which decreased NETosis triggered by A23187 or PMA (Fig. 2E,F). Thus, extracellular ATP may act in an autocrine and/or paracrine manner on purinergic signalling to modulate NETosis.

**Figure 3:**
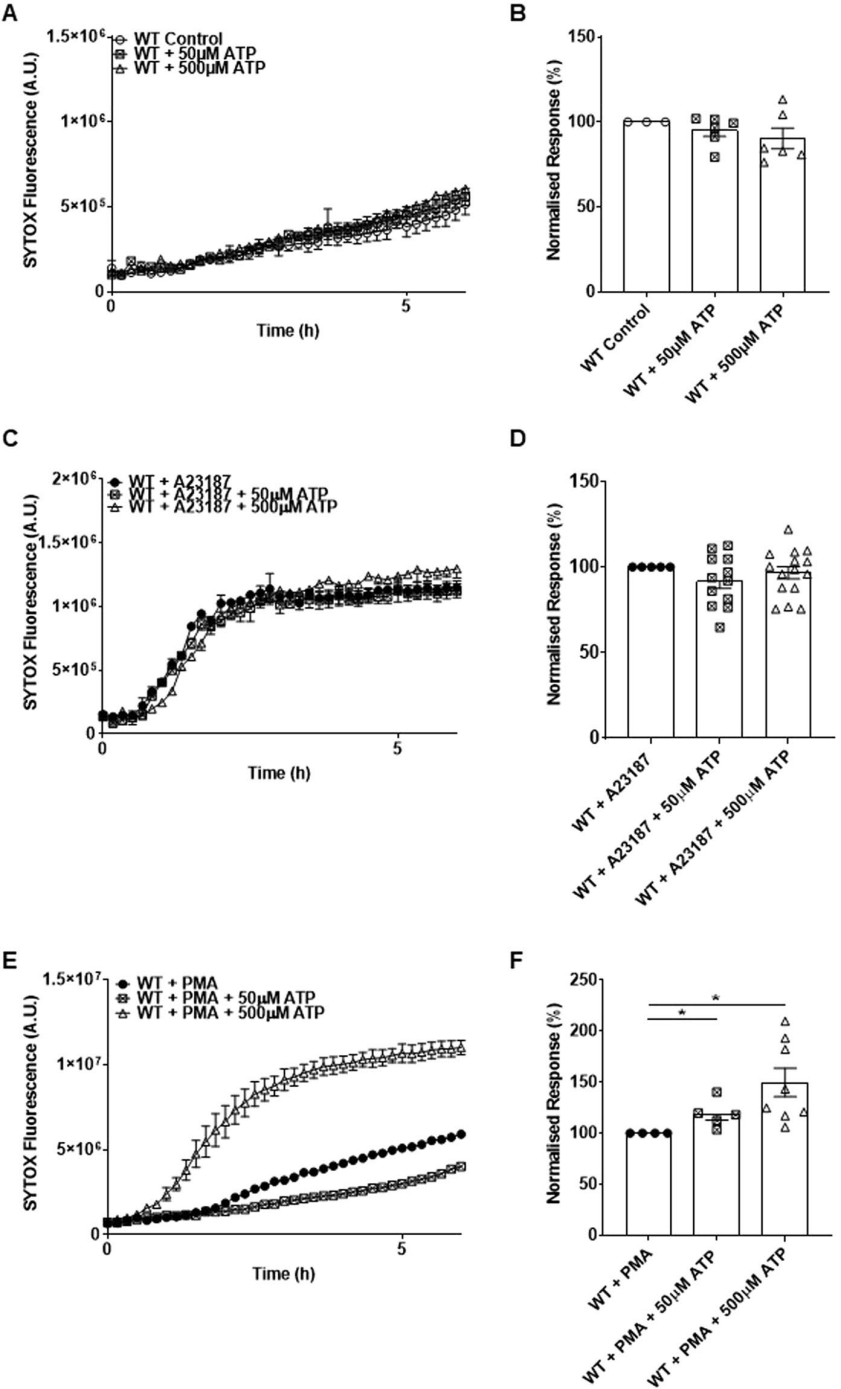
Role of ATP in the modulation of NETosis. (**A**) Kinetics of ATP stimulation (50 μM and 500 μM) in WT neutrophils at 6 h. (**B**) Quantification of ATP stimulation in WT neutrophils at 6 h. (**C**,**E**) Kinetics of ATP stimulation (50 μM and 500 μM) with A23187 and PMA stimulation respectively. (**D**,**F**) Quantification of response to ATP stimulation (50 μM and 500 μM) with A23187 and PMA stimulation respectively. (N ≥ 3). ^*^P < 0.05.

We next investigated whether ATP alone triggered NETosis. However, neither 50 μM nor 500 μM of extracellular ATP enhanced SYTOX fluorescence above that of controls (Fig. 3A,B). In addition, combining ATP and A23187 did not modify the time course or the amplitude of NETosis induced by A23187 alone (Fig. 3C,D), confirming that NETosis induced by the calcium ionophore was already maximal. Interestingly, we observed that adding ATP to PMA stimulation markedly enhanced NETosis, following a faster and more pronounced S-shaped kinetic similar to that observed for A23187 stimulation (Fig. 3E,F). Together, the data suggest that extracellular ATP is a modulator of NETosis, the rate and amplitude of which depend of the triggers used and of the purinergic signalling activated.

### Panx1 is functionally expressed in neutrophils

**Figure 4:**
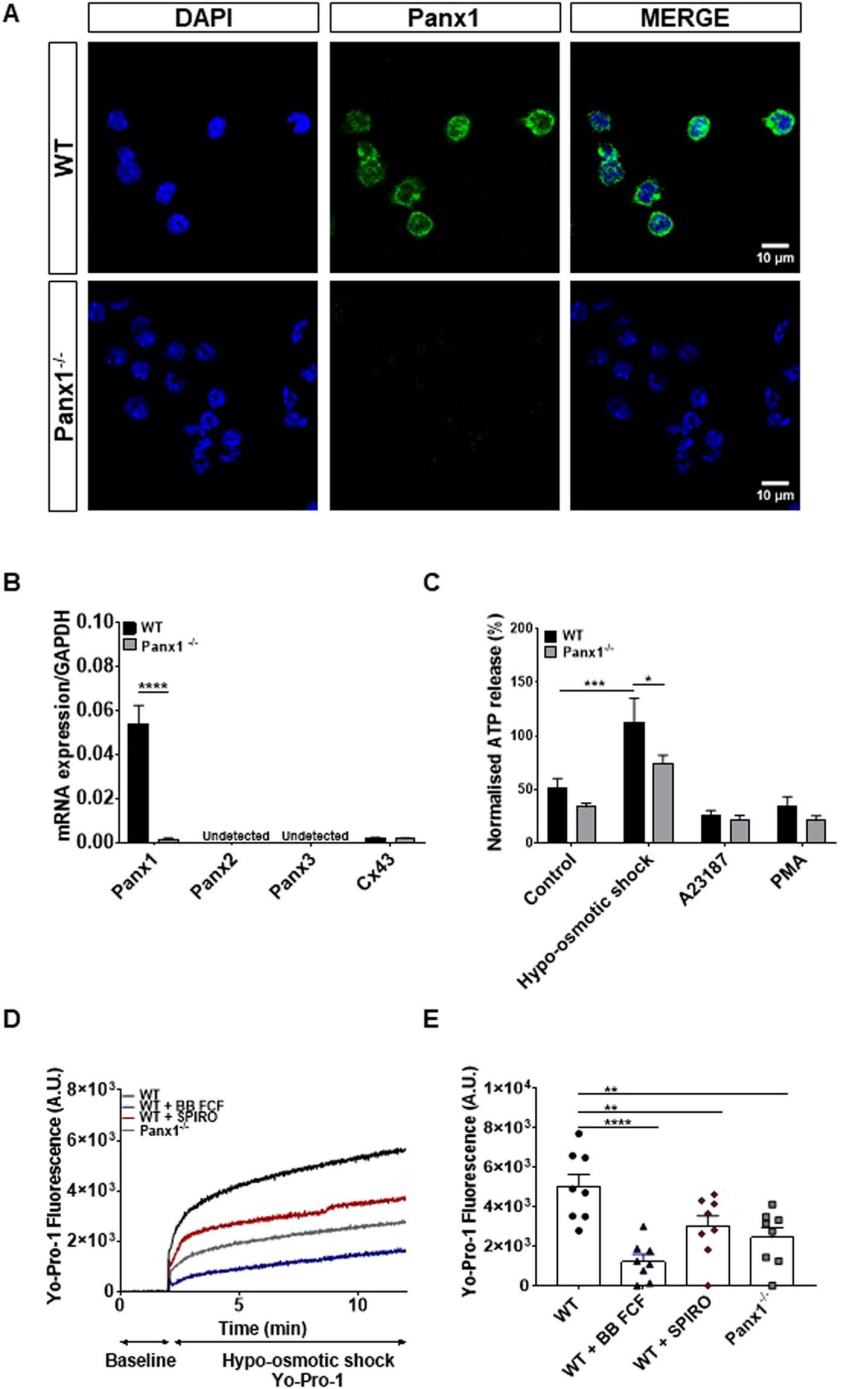
Panx1 expression and function in BMDNs. (**A**) Immunofluorescence of Panx1 (green), nuclei staining with DAPI (blue) in WT and *Panx1*^−/−^ BMDNs respectively. (**B**) mRNA expression levels of Panx1, 2, 3 and Cx43 in WT and *Panx1*^−/−^ BMDNs measured by qPCR (N = 3, n > 5). (**C**) Quantification of ATP release in WT and Panx1^−/−^ BMDNs with hypo-osmotic shock, A23187 and PMA stimulation at 5 minutes (N = 3, n = 6). (**D**) Representative kinetic of YO-PRO-1 dye uptake in the absence or presence of the Panx1 inhibitors BB-FCF (1 μM, blue) or spironolactone (100 μM, red) for WT BMDNs (black) as compared to *Panx1*^−/−^ BMDNs (grey). (**E**) Quantification of dye uptake in BMDNs (N = 4). ^**^P < 0.01; ^****^P < 0.0001.

Panx1, which forms ATP channels in neutrophils^19,20^, could contribute to ATP release during NETosis. Immunostaining of BMDNs confirmed Panx1 protein expression; Panx1 was mostly detected at the neutrophil membrane (Fig. 4A and Supplemental Fig. 2A). In contrast, Panx1 protein were absent in neutrophils isolated from *Panx1*^−/−^ mice (Fig. 4A). This was also confirmed at the mRNA (Fig. 4B) and at the protein (Supplemental Fig. 2B) levels. To exclude compensatory upregulation of other pannexin family members in *Panx1*^−/−^ BMDNs^22^, we verified for Panx2 and Panx3 transcripts, which were not detected in both WT and *Panx1*^−/−^ BMDNs (Fig. 4B). Connexin43 (Cx43) transcripts levels were very low and similar in both genotypes (Fig. 4B), excluding secondary effects from Cx43, which has been shown to form ATP releasing hemichannels in various cell types^20,23^. Panx1 function was first evaluated in WT and *Panx1*^−/−^ BMDNs by measurement of ATP release using hypo-osmotic shock (Fig. 4C). Within 5 min after stimulation, there was a marked increase in ATP release in WT neutrophils compared to *Panx1*^−/−^ neutrophils. Interestingly, A23187 or PMA stimulations did not elicit ATP release (Fig. 4C).

YO-PRO-1 dye uptake is another approach to monitor Panx1 function. Global measurement of YO-PRO-1 dye uptake in the overall neutrophil population was detected using a plate reader (Fig. 4D). BMDNs were first superfused with a control solution to acquire baseline fluorescence and YO-PRO-1 dye was then added to the medium. As a trigger to maximally open Panx1 channels, neutrophils were subsequently superfused by a hypotonic solution containing the same concentration of YO-PRO-1 dye. Increases in fluorescent signal were observed, the YO-PRO-1 fluorescence being markedly enhanced in WT as compared to *Panx1*^−/−^ neutrophils (Fig. 4D; black *vs*. grey curves). Importantly, YO-PRO-1 uptake in WT neutrophils decreased back to the level observed for *Panx1*^−/−^ BMDNs in the presence of the Panx1 inhibitors BB-FCF or spironolactone (Fig. 4D; black *vs*. blue and red curves, respectively). Quantification of the overall neutrophil population confirmed the reduction of YO-PRO-1 uptake in *Panx1*^−/−^ neutrophils and in the presence of Panx1 inhibitors (Fig. 4E). These results indicate that Panx1 is functionally expressed in BMDNs.

### NETosis is delayed in *Panx1*^−/−^ neutrophils and by Panx1 channel inhibition

**Figure 5:**
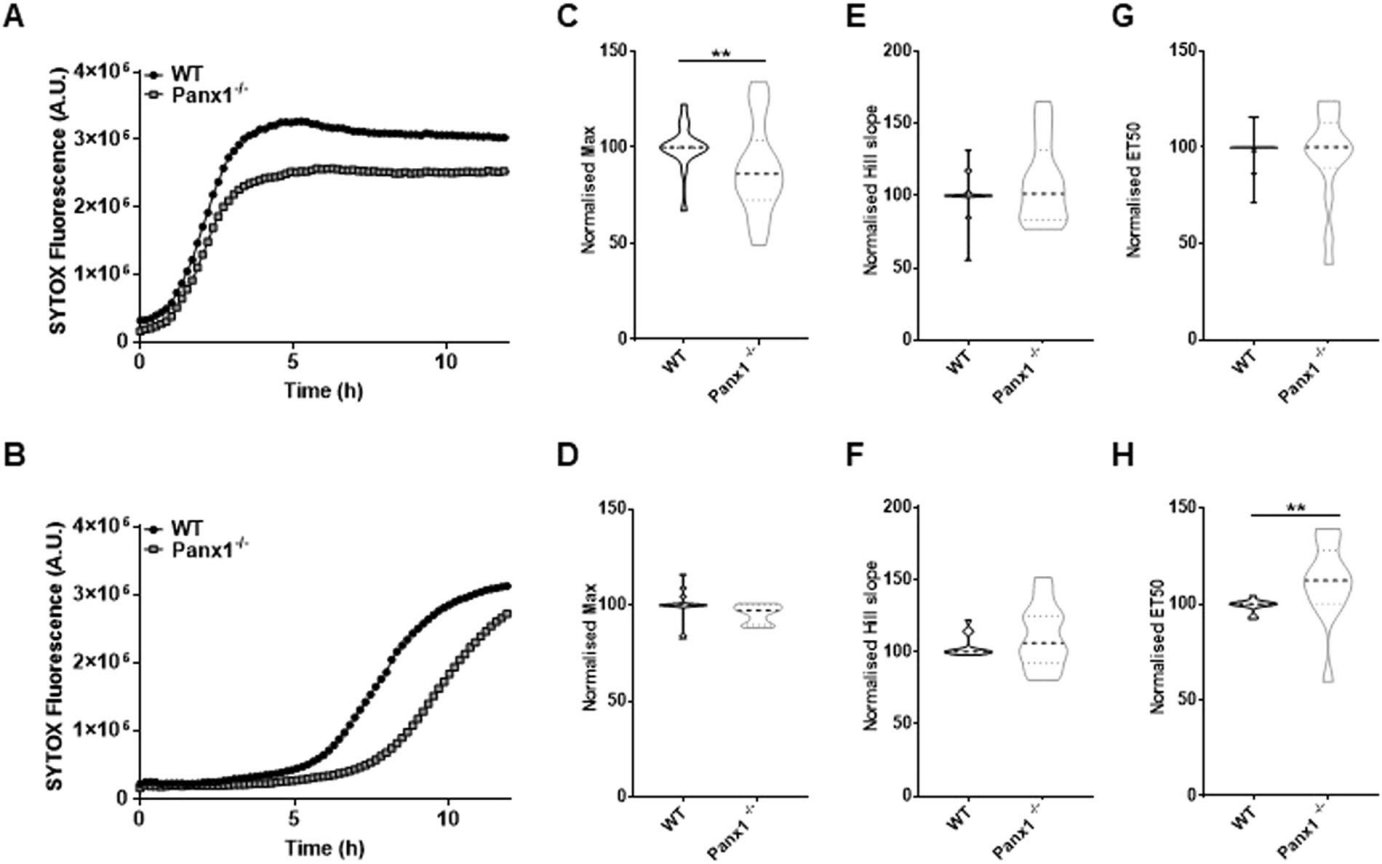
NADPH-dependent and independent NETosis in *Panx1*^−/−^ BMDNs. (**A**) Representative kinetic of A23187 (1 μM) stimulation in WT and Panx1^−/−^ neutrophils with SYTOX fluorescence over time. (**B**) Representative kinetic of PMA (50 nM) stimulation of WT and *Panx1*^−/−^ neutrophils with SYTOX fluorescence over time. (**C**,**D**) Quantification of Maximal (Max) values in A23187 and PMA stimulations respectively (n = 13). (**E**,**F**) Quantification of Hill Slope indicating rate of reaction in A23187 and PMA stimulations respectively (n = 13). (**G**,**H**) Quantification of half-maximal time (ET_50_) in A23187 and PMA stimulations respectively.

To determine if Panx1 channels contribute to NETosis, we compared the kinetics of extracellular DNA detection between WT and *Panx1*^−/−^ BMDNs exposed to A23187 (Fig. 5A) or PMA (Fig. 5B). For both stimuli, the kinetics of NETosis were slowed down in *Panx1*^−/−^ neutrophils. Using linear regression analysis, traces were fit to a sigmoidal curve and we obtained values for maximal response Max (Fig. 5C,D), Hill slope (Fig. 5E,F) and time to half maximal response ET_50_ (Fig. 5G,H) parameters. Violin plots of the distribution of normalised values are shown. For each experiment, the parameters obtained in *Panx1*^−/−^ BMDNs were normalised to that measured in WT neutrophils. Although no difference was observed for the Hill slope parameters, Max was decreased in *Panx1*^−/−^ neutrophils exposed to A23187, while ET_50_ was increased in *Panx1*^−/−^ neutrophils exposed to PMA. These results indicate that Panx1 contributes to NET formation and modulates the kinetic of NETosis according to the trigger used.

**Figure 6:**
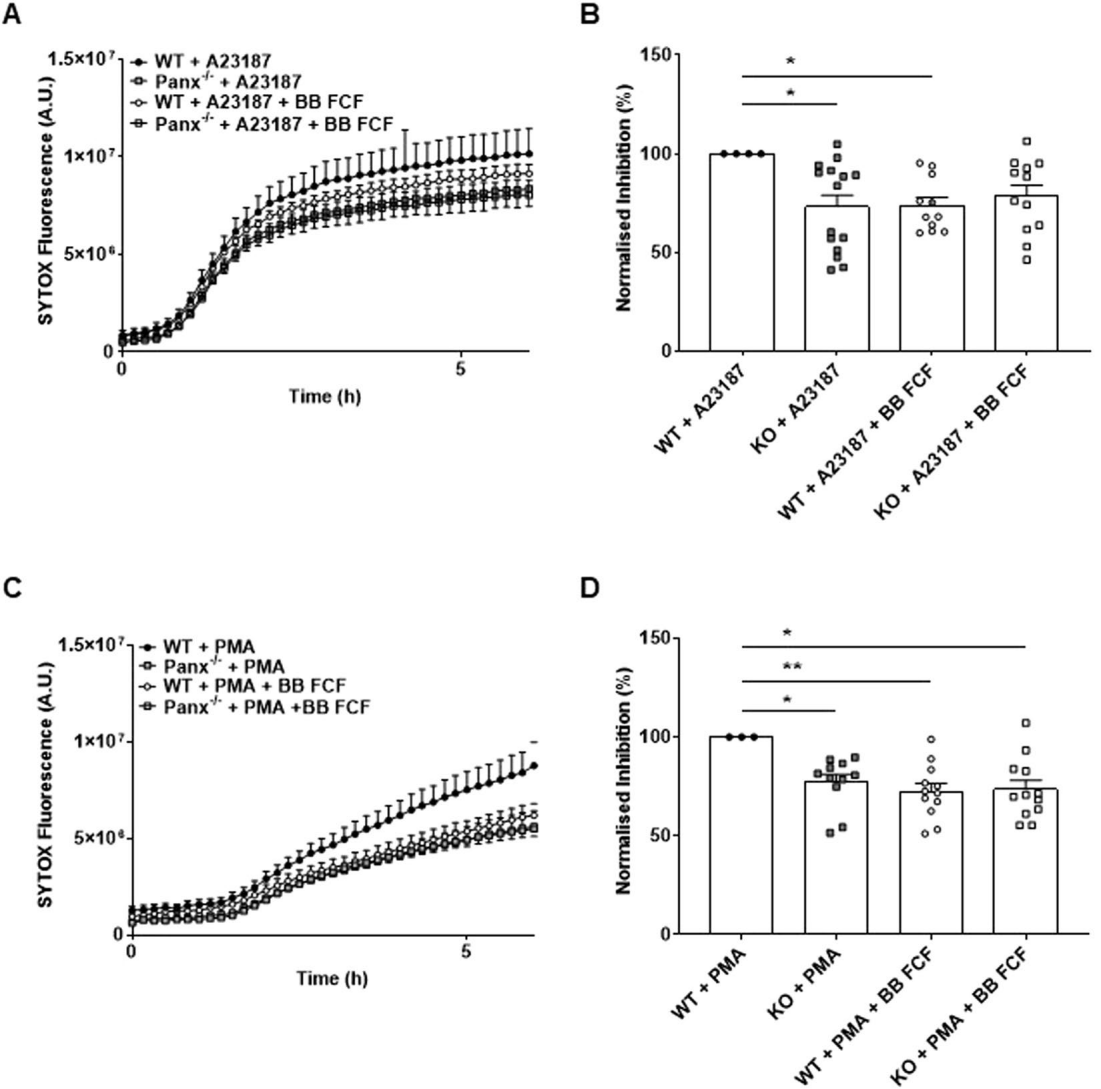
Chemical inhibition of Panx1 reduces NETosis in WT neutrophils. (**A**) Representative Kinetic of BB FCF dye (1 μM) inhibition of NETosis in WT neutrophils with A23187 stimulation. (**B**) Quantification of fluorescence measurements at 6 h (N = 4, n = 7). (**C**) Representative Kinetic of BB-FCF dye (1 μM) inhibition of NETosis in WT neutrophils with PMA stimulation. (**D**) Quantification of fluorescence measurements at 6 h (N = 3, n = 6) ^∗^P < 0.05; ^∗∗^P < 0.01.

BB-FCF has been shown to inhibit Panx1 channels in various cell types^24,25^, including neutrophils (Fig. 4D,E). Therefore, we evaluated the ability of the inhibitor to modulate the kinetic of NETosis in response to A23187 and PMA. We observed a delay in the kinetics in BB-FCF-treated WT neutrophils, which was comparable to the behaviour of *Panx1*^−/−^ BMDNs (Fig. 6A,C). After 6 h, comparison of the SYTOX fluorescence measurements showed significant reduction WT neutrophils exposed to BB-FCF (Fig. 6B). We observed similar effects in BB-FCF-treated WT neutrophils with PMA stimulation (Fig. 6D). These results show that Panx1 inhibitor BB-FCF reduced NETosis in WT BMDNs stimulated with both A23187 and PMA.

## Discussion

This study provides evidence that Panx1 and extracellular ATP, which is often elevated during inflammation, contribute to NETosis. Since the discovery of NETs^2^, NETosis is considered as a causal factor in the severity of various diseases. Whereas several intracellular signalling cascades have been identified, NETosis is clearly distinct from other processes causing cell death like apoptosis, necrosis and necroptosis^12^. Importantly, the initial phase of the process is associated with morphological changes and modifications of histones that prime the neutrophils for later mechanical changes. At this point, chromatin swelling is engaged leading to the irreversible rupture of the nuclear envelope and then the plasma cell membrane^13^.

A23187 and PMA are commonly used inducers of suicidal and vital NETosis. We confirmed the different dependence of these stimuli to NADPH oxidase and in the level of histone citrullination^5,21^. As expected, after several hours, both stimuli induced the release of DNA to the extracellular space that could be monitored by the use of the cell impermeable dye SYTOX green. The increase in SYTOX green fluorescence measured on a global population of stimulated BMDNs followed a sigmoidal curve. Consistent with video-microscopy recordings, the sigmoidal curve resulted from an increasing number of neutrophils undergoing NETosis with time. *Panx1*^−/−^ neutrophils were less sensitive to both A23187- and PMA-induced NETosis, yet different characteristics were observed. The number of *Panx1*^−/−^ BMDNs undergoing fast DNA release was reduced in response to A23187. However, DNA release was occurring with slower kinetics in *Panx1*^−/−^ than in WT neutrophils in response to PMA. Our data also shows decreased ATP release in *Panx1*^−/−^ neutrophils. Together, the results indicate that Panx1 accelerates the stimuli-triggered initial phase that leads to chromatin swelling, possibly by releasing ATP to the extracellular environment.

Purinergic regulation of neutrophil function is well established^19,20^. Extracellular ATP and its hydrolysed derivatives (ADP, AMP and adenosine) are critical signal transduction molecules, the balance of which is regulated by surface ectoenzymes. Thus, ATP and ADP are converted to AMP by ecto-apyrase CD39, which is highly expressed in neutrophils^26^. These nucleotides and nucleosides are ligands to P2 and P1 receptors respectively, which convert extracellular purine concentration into intracellular signals that modulate neutrophil immune response. Interestingly, one study reported that P1 receptor signalling could decrease NETosis induced by *Klebsiella pneumonia*^27^. The actual concentration of ATP close to the BMDN cell surface, which will vary with time, activity of ectoenzymes and number of activated purinergic receptors, is unknown. Nonetheless, we found that blocking P2Y_2_ receptors or scavenging ATP decreased NET formation, pointing to the P2 receptors as a major amplifier of NETosis. Extracellular ATP alone was not able to trigger NETosis, but enhanced NET formation evoked by PMA with kinetics similar to that obtained with A23187 stimulation. P2Y_2_ signalling has been previously involved in NET formation during acute phase of cattle neosporosis^28^. Also in line with these data is the report that uridine diphosphate (UDP), which is the natural ligand to P2Y_6_ receptor, fails to initiate NET formation in human neutrophils but UDP-mediated P2Y_6_ signalling is involved in the monosodium urate crystal-induced formation of NETs^29^. Together, these observations suggests that elevation of intracellular calcium concentration secondary to purinergic receptor activation plays a pivotal role in regulating the velocity of the NET response^21^.

Several mechanisms for ATP release from various cell types have been proposed, including vesicle exocytosis and channel-dependent transport among which connexons (made of connexins) and pannexons (made of pannexins) are extensively studied^30^. The release of ATP through Cx43-made connexons and Panx1 channels was previously reported in neutrophils in response to N-formylmethionyl-leucyl-phenylalanine, lipopolysaccharide or hypertonic saline^31,32^. Although Panx1 mRNA was detected in WT neutrophils, the levels of Panx2, Panx3 and Cx43 mRNA were negligible in both WT and *Panx1*^−/−^ BMDNs, excluding them from a possible compensatory mechanism. The molecular mechanism that opens Panx1 channels in neutrophils exposed to A23187 or PMA have not been examined here. It is worth noting that many cellular changes induced by A23187 and PMA during NETosis, including rise in intracellular calcium and cell swelling^13,21^, are also known triggers of Panx1 channel opening^33,34^. Thus, we propose that reduced ATP released by *Panx1*^−/−^ BMDNs may lower the ability of the nucleotide and its metabolites to activate purinergic receptors, thus increasing the delay before the point of no return. Once this point is reached and NETosis occurs in some BMDNs, ATP will be released due to membrane rupture, thus accelerating NETosis in the surrounding neutrophils by activating the purinergic signalling cascade. Importantly, we show here that pharmacological inhibition of Panx1 channels with BB-FCF is sufficient to decrease the number of neutrophils undergoing NETosis in response to A23187 and PMA.

The identification of the molecular pathways that contribute to chromatin swelling are relevant to identify therapeutic targets. BB-FCF is an FDA-approved blue dye used for food colouring and, possibly in combination with other purinergic receptor inhibitors, could represent an efficient mean to reduce detrimental NETosis, particularly under excessive inflammatory conditions. This is not only of relevance for (auto-) inflammatory diseases but also for cancer as NETs induced by inflammation could promote the awakening of dormant cancer cells^35^. Future work on animal models whereby Panx1 is selectively deleted from neutrophils will shed some light on the rational of targeting this channel in inflamed tissues.

## Materials and Methods

### Mice

The Swiss Federal veterinary authorities (“Autorité Cantonale, Direction Générale de la Santé”) approved all animal experimentation in accordance to established guidelines and regulations. NETosis experiments were performed using WT and *Panx1*^−/−^ mice on a C57BL/6 background. *Panx1*^−/−^ mice were previously characterised^36^. All experiments were performed using sex and age-matched animals. Mice were bred in the in-house animal facility and kept under standard housing conditions with fixed light/dark cycle.

### Neutrophil isolation

Neutrophils were isolated from bone marrow. Mice were killed, femurs and tibiae of the lower limbs were exposed and flushed to obtain bone marrow in calcium free PBS with EDTA (1 mM, Promega, #V4231) and fetal bovine serum (2%, ThermoFisher Scientific, #10270098). Neutrophil isolation and purification were carried out using EasySep™ negative selection mouse neutrophil enrichment kit and magnet, according to manufacturer’s protocol (Stemcell Technologies, #19762, #18000). Bone marrow derived neutrophils (BMDNs) were washed and maintained in RPMI 1640 without phenol red (ThermoFisher Scientific, #11835-063) supplemented with HEPES (10 mM, ThermoFisher Scientific, #15630056) prior to immediate use.

### Real-time PCR

BMDN RNA extraction was done using RNeasy mini kit (Qiagen, #74106). Genomic DNA removal and cDNA synthesis were performed using the RT Quantitect kit (Qiagen, #205311) with the UNOII PCR thermocycler (Biometra GmbH, Göttingen, Germany). qPCR was performed using TAQMAN gene primers (ThermoFisher Scientific, #Mm00450900_m1, #Mm01308054_m1, #Mm00552586_m1 and #Mm01179639_s1 for Panx1, Panx2, Panx3 and Cx43, respectively) with the Applied Biosystems StepOne Plus™ Real Time PCR system (ThermoFisher Scientific), according to the manufacturer’s pre-set amplification protocol. Gene expression was normalised to mouse glyceraldehyde 3-phosphate dehydrogenase (GAPDH) expression. Values were expressed as 2^−ΔCT^, according to the procedure described by Schmittgen and Lival, 2008^37^. Transcript detection with CT values above 34 were considered as undetected.

### Immunofluorescence

10^5^ BMDNs were seeded on coverslips by centrifugation in 24-well plate and were fixed with paraformaldehyde (4%) for 20 min at room temperature. Next, permeabilisation was performed with Triton-X100 (0.3%), neutralisation with ammonium chloride (0.5 M), and blocking with PBS containing bovine serum albumin (BSA; 2%). The chicken anti-mouse Panx1 (1:500), recognizing amino acids 414–425^38^, citrullinated histone 3 (1:300, Abcam, AB5103) and neutrophil marker NIMP-R14 (1/250, Santa Cruz, sc-59338) primary antibodies were then applied overnight. Goat anti-chicken (1:500) DyLight488 antibody (Jackson Laboratories), goat anti-rabbit (1:2000) Alexa 488 (Thermofisher Scientific) and IgG anti-rat (1:100) rhodamine secondary antibodies were then applied for 2 h at room temperature (RT) with DAPI counterstaining. Samples were mounted on coverslips with Vectashield (Vector Laboratories, #H-1000). Images were acquired using inverted Zeiss LSM700 laser-scanning confocal microscope (Carl Zeiss) through 10x, 20x, 40x or 63x oil immersion objectives. Quantification was performed using Fiji software. Histone H3 citrullination and DAPI fluorescence were quantified as area per image, while Panx1 fluorescence was quantified as integrated density per region of interest (ROI) in WT and Panx1^−/−^ BMDNs. Ratio values were expressed as arbitrary unit.

### Western blot

BMDNs and splenocytes from WT and Panx1^−/−^ mice were incubated in RIPA lysis buffer (1 mM PMSF, 0.2% Na-deoxycholate, 2 mM Na-orthovanadate, 10 mM NaF, 1X Roche Protease Inhibitor, 0.05% SDS, 1X NP40, 1 mM EDTA, pH 7.4.) for 15 minutes on ice. Samples were then centrifuged at 10’000 g and 4 °C for 15 minutes. Proteins were quantified by Pierce bicinchoninic acid (BCA) assay (ThermoFisher Scientific). 50 µg and 10 µg of total protein from BMDNs and splenocytes, respectively, were separated on a sodium dodecyl sulphate (SDS) polyacrylamide gel and transferred on nitrocellulose membrane. Membrane was blocked in PBS-Tween (0.01%-PBST) buffer containing 5% defatted milk for 1 hour at RT with agitation. Monoclonal Panx1 (D9M1C Cell Signalling, 91137) antibodies were diluted 1: 1000 in 5% milk in PBST for overnight incubation at 4 °C, followed by 2 hours incubation at RT with goat anti-rabbit horse radish peroxidase (HRP)-conjugated antibody (Jackson ImmunoResearch Laboratories). Revelation was performed using WesternBright™ Sirius™ (Advansta). Total amount of loaded protein was verified using Ponceau red staining.

### Panx1 functional assay

For ATP release assay, BMDNs were left to equilibrate in Tyrode’s buffer (124 mM NaCl, 2.4 mM KCl, 10.8 mM NaHCO_3_, 0.4 mM NaH_2_PO_4_*H_2_O, 0.9 mM MgCl_2_*6H_2_O, 1.8 mM CaCl_2_*6H_2_O, pH 7.35). Panx1 channel mediated ATP release was activated by reducing osmolarity of the Tyrode’s buffer by decreasing NaCl to 30.24 mM. WT and *Panx1*^−/−^ BMDNs were stimulated with appropriate controls for 5 min. Cells were centrifuged at 1200 rpm for 5 minutes and supernatants were collected for ATP measurement using the ATP bioluminescent assay kit (#FLAA, Sigma Aldrich), according to the manufacturer’s instructions.

For dye uptake assay, BMDNs were seeded in 384-well plate and maintained in a physiological solution (136 mM NaCl, 4 mM KCl, 1 mM CaCl_2_, 1 mM MgCl_2_, and 2.5 mM glucose, buffered to pH 7.4 with 10 mM HEPES-NaOH solution). YO-PRO-1 Iodide (5 µM, ThermoFisher Scientific, #Y3603) was added to equilibrate cells. Hypo-osmotic shock was induced to maximally open Panx1 channels. Inhibitors of Panx1 BB-FCF dye-Erioglaucine disodium salt (Sigma Aldrich, #80717)^24,25^ and spironolactone (Sigma Aldrich, #S3378)^39^ were prepared in DMSO and used at 1μM and 100 μM, respectively. Global quantification of YO-PRO-1 dye uptake per well was performed using the FDSS/µCELL Functional Drug Screening System (Hamamatsu Photonics).

### *In Vitro* quantification of NET formation

NETosis measurements were made as follows; 10^5^ BMDNs were seeded in 96-well assay plate (Corning #3603, Sigma Aldrich). 50 nM PMA or 1 μM A23187 (Sigma Aldrich, #P1585, #C7522 respectively) were added to wells in duplicates/triplicates with DMSO control included. The cell impermeable SYTOX green nucleic acid stain (5 µM, ThermoFisher Scientific #S7020) was added. SYTOX green stain measurement was performed using Spark Microplate Reader (Tecan) fitted with monochromatic filter 504/523 nm. 1 μM BB-FCF was used for Panx1 inhibition with appropriate controls. ATP scavenger apyrase grade VII (50 U/ml, Sigma Aldrich, #A6535), P2Y_2_ receptor antagonist AR-C 118925XX (500 nM, Tocris, #4890) and CD39 ARL 67156 trisodium salt (100 μM, Tocris, #1283) were used for the purinergic signalling experiments involving ATP release. 10 mM N-acetyl cysteine (NAC, Sigma Aldrich #A0737) was used for ROS inhibition. For time-lapse imaging, SYTOX green dye (5 μM) was added to BMDNs seeded in 96-well assay plate. Cells were stimulated with 1 μM A23187 for 30 min in the presence of appropriate controls. Images were acquired at 10 minutes intervals for 5 h using the ImageXpress Micro Widefield High Content Screening System (Molecular Devices, San Jose, USA). MetaXpress 2.0 software was used to reconstitute images for video analysis.

### Statistics

Statistical analyses were performed using Prism 8.0.2 (Graph Pad Prism). Data was expressed as mean ± SEM. Nonlinear regression analysis was performed using the log (agonist) vs. response- Variable slope (four parameters) for SYTOX fluorescence traces. Background fluorescence was subtracted and the maximal fluorescence value (MAX), Hill slope and the time (ET50) for response halfway between the basal (Min) value and the maximal (Max) value were determined. One-way ANOVA and non-parametric Student t test (Mann-Whitney) were used when appropriate and *P ≤ 0.05, **P ≤ 0.01, ***P ≤ 0.001, ****P ≤ 0.0001 were considered significant.

## Supplementary information

Supplementary Information

Supplemental Video 1
