# Supplementary material for: ATP amplifies NADPH-dependent and -independent neutrophil extracellular trap formation
Source: Sci Rep. 2019 Nov 12;9:16556. doi: 10.1038/s41598-019-53058-9 (PMC6851112; doi:10.1038/s41598-019-53058-9)
Supplement: Supplementary file 1 — Supplementary Information [file 41598_2019_53058_MOESM1_ESM.pdf]

ATP amplifies NADPH-dependent and -independent neutrophil extracellular trap formation

Aderonke Sofoluwe[1,2], Marc Bacchetta[1,2], Mehdi Badaoui[1,2],

Brenda R. Kwak[3], Marc Chanson[1,2*]

[1]*Department of Paediatrics, Gynaecology & Obstetrics, Geneva University Hospitals, Geneva, Switzerland*

[2]*Department of Cell Physiology & Metabolism, University of Geneva, Faculty of medicine, Geneva, Switzerland*

[3]*Department of Pathology & Immunology, University of Geneva, Faculty of medicine, Geneva, Switzerland*

[*]Corresponding author:

Marc Chanson PhD

University of Geneva / Centre Médical Universitaire (PHYM)

1, Rue Michel-Servet

1211 Geneva (Switzerland)

Tel: +41 22 37 95 206; **Fax:** +41 22 37 95 260

Email: Marc.Chanson@unige.ch

Supplemental Video 1. Time-lapse imaging of YO-PRO-1 fluorescence in BMDNs exposed to 1 μM A23187. Recording starts after 30 min of stimulation.

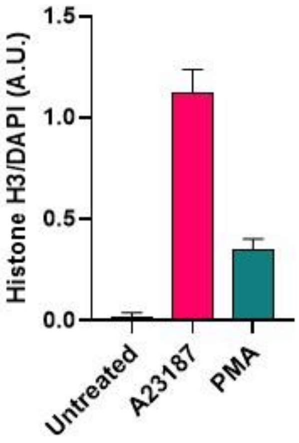

Supplemental Figure 1. Quantification of citrullinated histone immunofluorescence induced with 1 μM A23187 or 50 nM PMA for 2h as compared to untreated neutrophils (n=3).

A

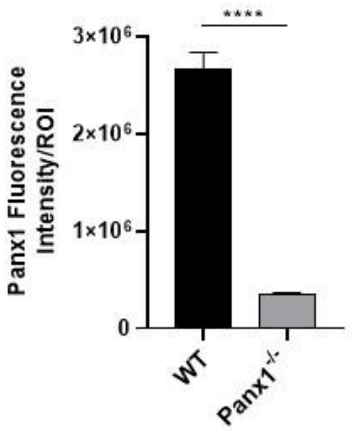

B

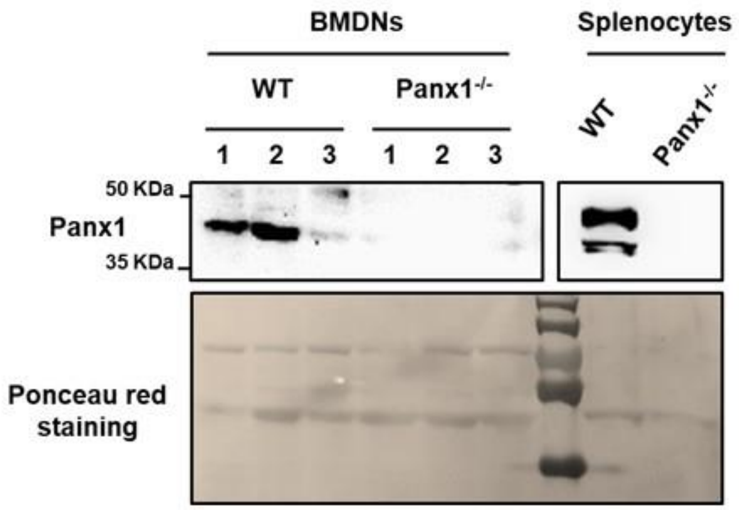

Supplemental Figure 2. Expression of Panx1 in mouse BMDNs. **A)** Quantification of immunofluorescence images shown in Figure 4A. **B)** Western blot showing the expression of Panx1 in mouse BMDNs and splenocytes, which were used as positive and negative controls. Panx1 was detected in 3 WT BMDN samples (lines 1-3) but not in 3 Panx1$^{-/-}$ BMDN samples (lines 1-3) (top panels). Note that the exposure time to reveal Panx1 was different between BMDNs and splenocytes. Ponceau red staining of the immunoblot membrane showing total amount of protein loaded (bottom panel).

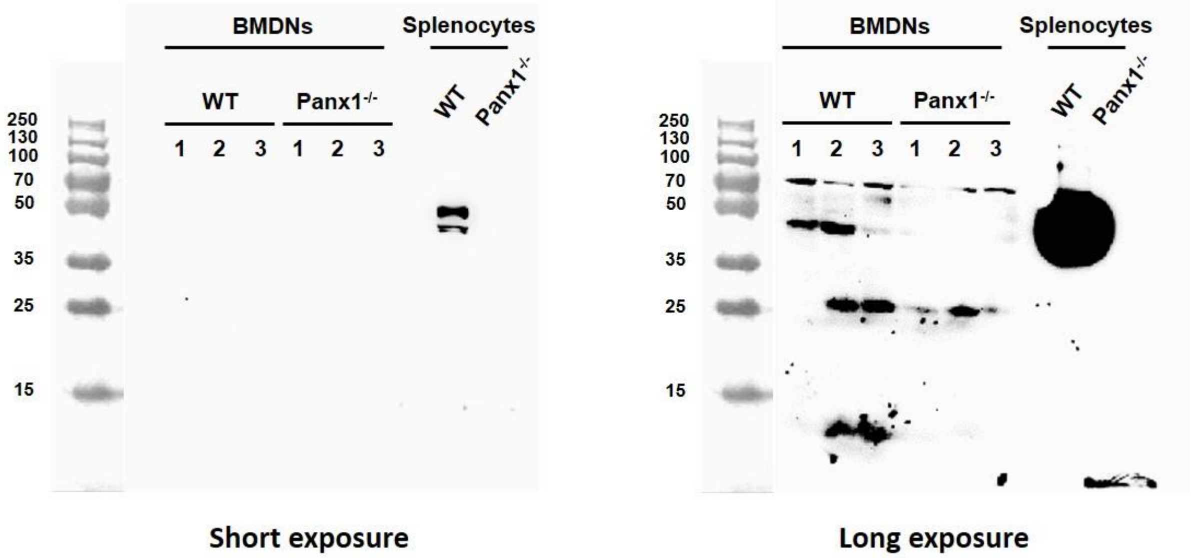

Supplemental Supplemental Figure 2B. Full-length Western blots shown in Supplemental Figure 2B. Short (left panel) and long (right panel) exposure time of the membrane. For creating Supplemental Figure 2B, the long exposed membrane image was cropped between 35 and 50 kDa molecular weights (shown at the left side of the panels). For positive and negative control splenocytes, the short exposed membrane image was used.
